# Diagnostic Challenges and Therapeutic Considerations in Adult-Onset Still’s Disease: A Clinical Case Report

**DOI:** 10.7759/cureus.74054

**Published:** 2024-11-19

**Authors:** Muhammad Wasim Tariq, Muhammad Tanseer Sibtain Raza, Reefat Farzina, Sathia Narayanan Mannath, Cornelius J Fernandez

**Affiliations:** 1 General Medicine and Endocrinology, United Lincolnshire Hospitals NHS Trust, Boston, GBR; 2 Critical Care, Nottingham University Hospitals NHS Trust, Nottingham, GBR; 3 Gastroenterology, United Lincolnshire Hospitals NHS Trust, Boston, GBR; 4 Diabetes and Endocrinology, United Lincolnshire Hospitals NHS Trust, Boston, GBR

**Keywords:** adult-onset still’s disease, fautrel criteria, hyperferritinemia, multi-organ failure, systemic inflammatory disorder, yamaguchi criteria

## Abstract

Adult-onset Still's disease (AOSD) is an uncommon systemic inflammatory disorder that presents with diverse, overlapping symptoms, complicating the diagnostic process due to its nonspecific clinical features and the absence of a definitive diagnostic test. Diagnosis is often challenging and relies on excluding other conditions while maintaining a high index of suspicion, supported by specific diagnostic criteria such as Yamaguchi or Fautrel. Prompt recognition and a multidisciplinary approach are essential, as AOSD can progress to life-threatening multiorgan dysfunction due to a hyperinflammatory response.

We report the case of a 58-year-old male car mechanic with no prior medical history who presented with a three-month history of persistent fever, bilateral hip pain, and systemic symptoms. On examination, he was febrile (39.4°C) with tachycardia, tachypnea, and elevated inflammatory markers with eosinophilia. Despite a comprehensive workup, including imaging and broad-spectrum antibiotics, his condition progressed, marked by the emergence of a characteristic salmon-pink rash, altered mental status, and persistent fever. Significant findings included markedly elevated ferritin levels (43,980 ng/mL), normal creatine kinase, negative autoimmune markers, and PET-CT revealing fluorodeoxyglucose (FDG)-avid intramuscular sites. A muscle biopsy demonstrated perivascular lymphocytic infiltration. A diagnosis of AOSD was established, and high-dose corticosteroids were initiated, leading to initial improvement. However, the patient developed complications, including acute kidney injury, steroid-induced diabetes, resistant hypertension, and cardiopulmonary symptoms, eventually succumbing to acute respiratory failure six weeks post-discharge. This case underscores the severe potential trajectory of AOSD and the complex diagnostic and therapeutic challenges associated with its management.

## Introduction

Adult-onset Still's disease (AOSD) is a chronic systemic inflammatory disorder of unknown etiology. AOSD is characterized by a high spiking fever, sore throat, myalgia, polyarthralgia, salmon-colored evanescent rash, hepatosplenomegaly, lymphadenopathy, and neutrophilic leukocytosis [1]. AOSD has a bimodal age distribution across all ethnic groups and both sexes, with peaks at 15-25 years and 36-46 years of age. It is one of the rare inflammatory disorders with a global incidence of 0.16 and 0.62 per 100,000 individuals [2].

AOSD poses unique challenges in diagnosis due to its non-specific symptoms that can overlap with other conditions. The disease can range from mild and self-limiting to severe and chronic with potentially fatal complications if left untreated.

The treatment for AOSD comprises anti-inflammatory medicines like non-steroidal anti-inflammatory drugs (NSAIDs) and corticosteroids for mild to moderate disease. However, for moderate to severe disease, the use of disease-modifying antirheumatic drugs (DMARDs), biological agents (e.g., IL1-Ra anakinra), or intravenous immunoglobulins can also be considered.

## Case presentation

A 58-year-old gentleman, working as a car mechanic, with no significant past medical history, presented with a three-month history of fever and malaise. He had made multiple emergency department visits, receiving oral antibiotics without significant improvement. On his fourth presentation, he reported an eight-week history of bilateral hip pain, right lower limb pain leading to decreased mobility, and an 11-pound weight loss with night sweats.

An initial examination of his lower limb revealed an inability to move his right hip with tenderness at the lateral aspect. At presentation, his observations were as follows: pulse 117 bpm, BP 150/93 mmHg, respiratory rate 30/min, temperature 39.4℃ with SpO_2_ 94% in room air. Initial investigations revealed Hb 139 g/L, raised inflammatory markers (CRP: 92 mg/L, WCC: 13.8 x 10^9/L, neutrophils: 10.88 x 10^9/L), and eosinophilia (1.15 x 10^9/L). Urea, electrolytes, and liver function tests are normal except for low albumin, 2.1 gm/dl. Arterial blood gas revealed a lactate level of 1.9 mmol/L, with no evidence of metabolic acidosis. The D-dimer was elevated at 882 ng/mL. A computed tomography (CT) pulmonary angiogram (CTPA) excluded pulmonary embolism. A further CT of the chest, abdomen, and pelvis (CT CAP) done to evaluate weight loss demonstrated findings indicative of mesenteric panniculitis without any gross hepatosplenomegaly. Venous Doppler ultrasound of the right leg ruled out deep vein thrombosis (DVT).

**Figure 1:**
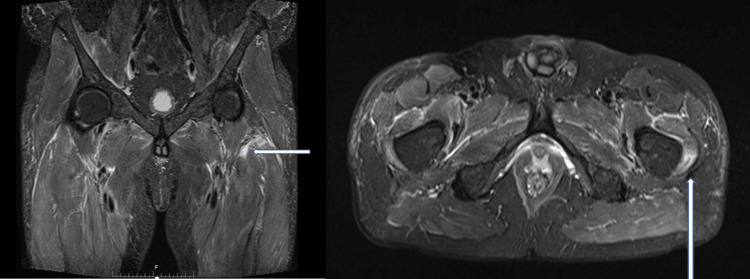
MRI pelvis Focal edema involving the left vastus lateralis and distal iliopsoas muscles (an area of hyperintensity indicating muscle edema highlighted by arrows)

The patient was initially treated for sepsis with intravenous cefuroxime and metronidazole. Analgesics were added for the hip and lower limb pain. An opinion from the gastroenterology and general surgery teams was sought to evaluate mesenteric panniculitis, which was deemed clinically insignificant, further delaying the diagnostic process as it can mimic other conditions such as infections, malignancies, or autoimmune diseases involving the abdomen. Following orthopedic consultation for chronic hip pain, an MRI of the pelvis was done, which revealed focal edema involving the left vastus lateralis and distal iliopsoas muscles, raising the concern of an ongoing systemic inflammatory or autoimmune process (Figure 1). Based on these findings, rheumatology opinion was consulted, and a series of investigations like rheumatoid factor (RF), cyclic citrullinated peptide (CCP), antinuclear antibody (ANA), antineutrophil cytoplasmic antibody (ANCA), complement 3 (C3), complement 4 (C4), and cardiolipin antibody were done, yielding negative results, leading to consideration of ordering a PET scan, specifically looking for vasculitis.

Urine dipstick analysis revealed 2+ proteinuria and 3+ hematuria, with a urine protein-to-creatinine ratio (PCR) of 177. The renal team was satisfied with the results of the vasculitis screen and additionally requested a myeloma screen, which returned normal. During ongoing investigations, intravenous meropenem was administered for presumed sepsis of unknown origin based on microbiology recommendations, given persistently elevated infection markers and lack of response to previous treatment, although repeated blood cultures were negative. He had an ongoing fever that started to become more frequent with spikes, developing a characteristic pink erythematous macular evanescent rash on the trunk over the course of the disease, and altered consciousness level during the spikes with increased breathlessness.

A chest X-ray performed due to breathlessness revealed pulmonary congestion. NT-Pro-BNP was elevated at 643 pg/mL, while echocardiography demonstrated a preserved ejection fraction (>55%) with no valvular vegetation. Inflammatory markers continued to rise, with a peak CRP of 332 mg/L, a white cell count of 19.2 x 10^9/L, and a platelet count of 588 x 10^5/L. Notably, there was sustained eosinophilia reaching 10.65 x 10^9/L, which rapidly decreased to 0.05 x 10^9/L after administration of 100 mg hydrocortisone during a medical emergency call for a suspected antibiotic allergy or drug reaction, triggered by an erythematous rash accompanying fever spikes.

**Figure 2:**
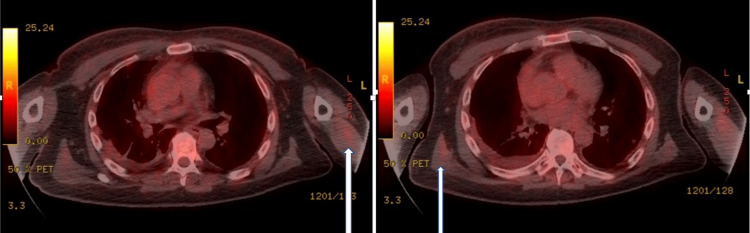
PET scan The unusual pattern of distribution of FDG-avid intramuscular foci, within the upper arms, shoulder girdle, as well as the posterior chest (highlighted by arrows) FDG: fluorodeoxyglucose

By this time, as he had been on IV meropenem for a month, our differential diagnoses were either lymphoma or vasculitis with antibiotic-induced eosinophilia. The PET scan was done, which revealed an unusual distribution of FDG-avid intramuscular foci in the shoulder girdle pointing toward a radiological diagnosis of myositis (Figure 2). However, the creatine kinase levels were normal throughout, whereas lactate dehydrogenase (LDH) was mildly raised at 928 U/L. Moreover, ferritin levels were markedly raised, measuring 43,980 ng/mL. Blood film (peripheral smear) showed no Reed-Sternberg (RS) cells or any other significant pathology. A muscle biopsy was done, which showed perivascular lymphocytic infiltration without associated vasculitis changes.

In the interim, the patient’s condition continued to deteriorate, evidenced by a decreased Glasgow Coma Scale score, persistent fever, a fall from bed resulting in right upper limb weakness, and a left gaze preference, though CT head excluded any central cause. An urgent lumbar puncture was done as per neurology's suggestion, which was inconclusive. All the CSF viral screens were negative. A follow-up rheumatology review suggested managing the patient with a working diagnosis of ANCA-negative vasculitis with concurrent myositis as part of the differential diagnosis. The patient received intravenous methylprednisolone 500 mg for three days, followed by prednisolone at a dose of 1 mg/kg to be tapered by 10 mg fortnightly to reach a maintenance dose of 10 mg. The patient responded immediately; the fever subsided, he became more alert, and his inflammatory markers as well as eosinophilia came down. His ferritin level dramatically went down to 2596 ng/ml within two weeks, suggesting a systemic inflammatory process rather than sepsis. Subsequently, the diagnosis was revised to AOSD considering the characteristic fever pattern, salmon-colored rash, leukocytosis, elevated ferritin levels, and steroid responsiveness, and discharged on a tapering dose of steroid therapy following a hospital stay of 40 days.

**Figure 3:**
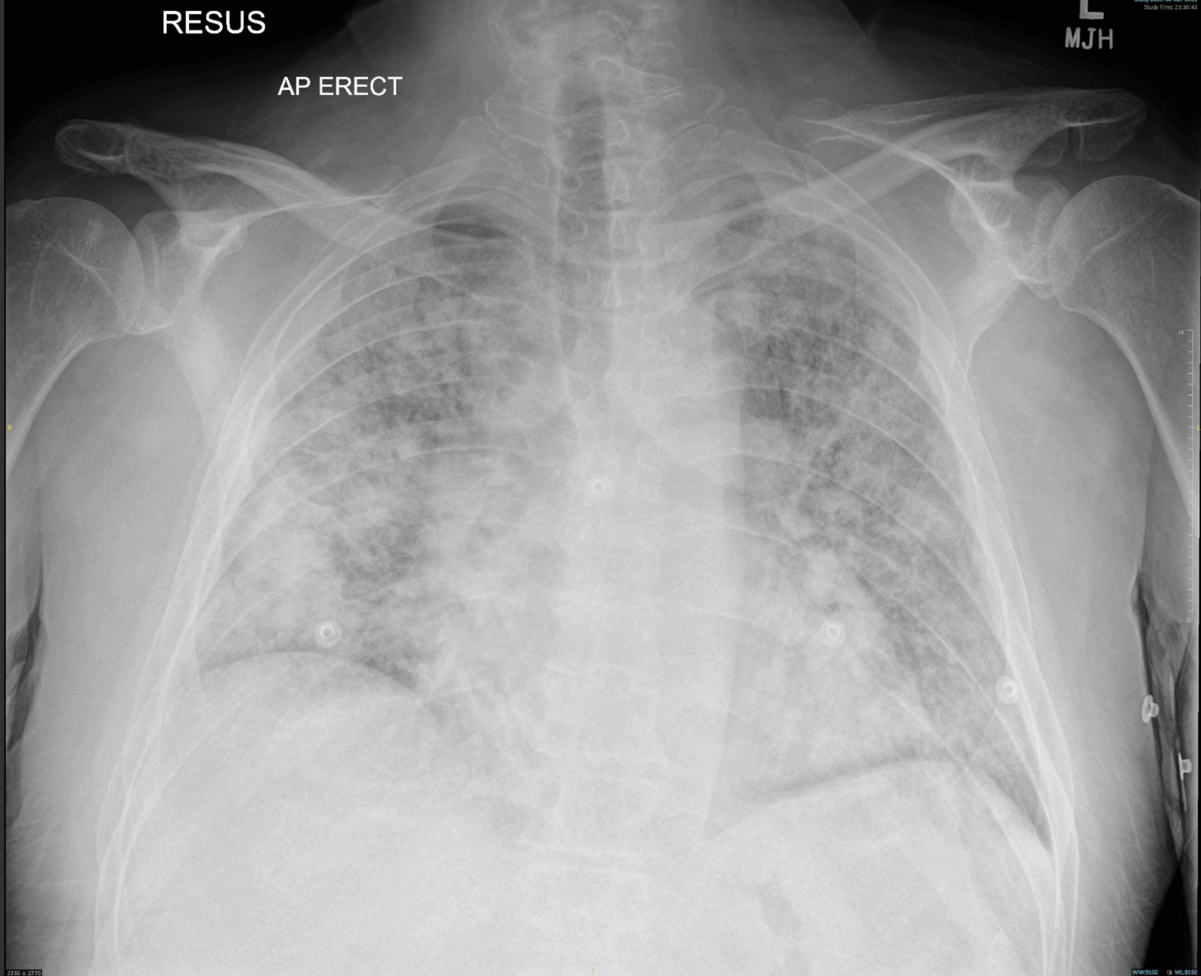
Chest X-ray Extensive bilateral patchy airspace opacifications suggestive of pneumonia, superadded by pulmonary edema

The patient was re-admitted after two weeks, with acute kidney injury stage 3 (eGFR dropping from >90 mL/min to 23 mL/min), steroid-induced diabetes, resistant hypertension, and hospital-acquired chest infection. His NT-ProBNP was 11,011, and repeat echocardiography revealed an EF above 55%, mild right ventricular systolic impairment, and an intermediate probability of pulmonary hypertension. A renal biopsy was performed but had to be abandoned due to bleeding complications, requiring embolization. The patient responded well to broad-spectrum antibiotics for hospital-acquired pneumonia, which could probably be attributed to the immune system malfunctions associated with AOSD. The patient was discharged but re-presented to the emergency department three weeks later with acute dyspnea. Chest X-ray was reported as extensive bilateral patchy airspace opacifications suggestive of pneumonia, superadded by pulmonary edema (Figure 3). Despite medical intervention, the patient deteriorated and succumbed to the illness the following day. Retrospectively, the acute presentation with rapid deterioration in this patient could be due to the macrophage activation syndrome (MAS) in the background of AOSD.

## Discussion

AOSD is a very rare systemic inflammatory disorder. The global incidence of AOSD is between 0.16 and 0.62 per 100,000 individuals [2]. It is characterized by the classical triad of fever, arthralgia/arthritis, and a salmon-colored transient rash; all of which were observed in our patient. A similar triad is also described in a pediatric condition known as systemic juvenile idiopathic arthritis (SJIA) which was described initially by Still in 1897 [3]. When a similar presentation was noted in adults, the disease is called Adult-onset Stills disease, which was first described by Eric Bywaters in the early 1970s [1].

Although the exact cause of AOSD is unknown, aberrant activation of innate immune cells, such as neutrophils, monocytes, and macrophages, as well as excessive cytokine production, including IL-1β, IL-6, TNF-alpha (tumor necrosis factor-alpha) and IL-18 are likely to be significant contributing factors [4]. Genetic factors (HLA DRB1*1201 and 1501, B35, DR2, and DR5), viral infections, bacterial infections, and stressful life events are all proposed as likely triggers [5].

Clinical presentation is usually non-specific and can vary among patients, with some cases being presented only with a fever of unknown origin (FUO). Associated arthritis affects the wrist joint, knee joint, ankle joint, and sometimes proximal interphalangeal joint and metatarsophalangeal joints. The diverse range of presenting clinical symptoms can contribute to delays in the diagnosis of the condition. The rash typically manifests on the trunk and proximal limbs and is transient, occurring primarily during fever spikes, often in the late afternoon or evening. In this patient, this characteristic presentation guided further diagnostic investigations and narrowed down the potential causes. It is often misdiagnosed as drug rash. Koebner phenomenon and dermatographism are reported in some [6]. Although myalgia is common in AOSD, myositis is rare.

The most often used criteria for the diagnosis of AOSD among the various criteria available is the Yamaguchi criteria (Table 1), which was proposed in 1992 [7]. The diagnostic criteria include four major, five minor, and three exclusion criteria. In 2002, Fautrel et al. suggested a revised criterion (Table 2) that incorporated two new markers including serum glycosylated ferritin ≤20% and ≥80% polymorphonuclear neutrophil count [8]. Applying the criteria, the patient met all major criteria, with negative PF and ANA tests. Extensive investigations also ruled out malignancy, sepsis, and vasculitis during the diagnostic workup.

**Table 1:** Yamaguchi’s criteria Must meet ≥5 criteria, of which ≥2 must be major. Reference: [7] WBC: white blood corpuscles; PMN: polymorphonuclear cells; LFT: liver function test; RF: rheumatoid factor; ANA: antinuclear antibody

Major criteria	Minor criteria	Exclusion criteria
Fever > 39^0^C > 1 week	Sore throat	Sepsis
Arthralgia/arthritis > 2 weeks	Lymphadenopathy	Malignancy (lymphoma)
Typical rash	Hepatosplenomegaly	Vasculitis
WBC > 10,000, 80% PMN’s	Deranged LFTs	
	RF and ANA negative	

**Table 2:** Fautrel’s criteria Must meet ≥4 major or 3 major plus 2 minor criteria. Reference: [8] WBC: white blood corpuscles; PMN: polymorphonuclear cells; GF: glycosylated ferritin

Major criteria	Minor criteria
Fever spikes > 39^0^C	Maculopapular rash
Arthralgia	WBC >10,000
Evanescent erythema	
Pharyngitis	
PMN ≥80%	
GF ≤20%	

In addition to the classical triad, AOSD can present with weight loss, hepatomegaly, splenomegaly, lymph node enlargement, raised neutrophil count, pleuritis, pericarditis, pneumonitis, and renal impairment. Renal involvement is not so common in AOSD. Various mechanisms of acute kidney injury in AOSD include mesangial proliferative glomerulonephritis, disseminated intravascular coagulation, thrombotic microangiopathy, collapsing glomerulopathy, and renal amyloidosis [9]. In our patient, recurrent presentations may have been associated with one of these renal pathologies, although a definitive diagnosis could not be established due to the unsuccessful attempt at renal biopsy. Cardiac involvement in AOSD can present with pericarditis, cardiac tamponade, myocarditis (presenting as heart failure), fulminant myocarditis (presenting as cardiogenic shock), non-infective endocarditis, and pulmonary arterial hypertension [10].

Ferritin levels increased in the majority with AOSD, with ≥35% having levels above 1000 ng/mL and ≥20% having levels above 3000 ng/mL. Ferritin might reflect an acute-phase response. Alternatively, it might be playing a role in the AOSD pathogenesis. In either case, raised ferritin levels help in making the diagnosis, especially in the presence of other features [11]. Moreover, markedly raised ferritin levels are observed in MAS (Table 3) [12], a secondary form of hemophagocytic lymphohistiocytosis (HLH), seen in autoimmune conditions including AOSD, with other causes being SJIA, systemic lupus erythematosus, systemic sclerosis, Kawasaki disease, spondyloarthropathy, dermatomyositis, and inflammatory bowel disease [13].

**Table 3:** Diagnostic criteria for MAS Reference: [12] MAS: macrophage activation syndrome; TG: triglyceride; AST: aspartate transaminase

MAS - diagnostic criteria
Fever AND
Ferritin > 684
Any two out of four of the following
Platelets ≤ 181 × 10^9^/L
Fasting TG > 156 mg/dL
AST > 48 U/L
Fibrinogen ≤ 360 mg/dL

MAS occurs in 7-15% of AOSD. The diagnosis of MAS in AOSD is challenging, as AOSD and MAS have many clinical and laboratory findings in common, including fever, hepatomegaly, lymphadenopathy, splenomegaly, and raised ferritin levels [14]. A specific set of diagnostic criteria exists for MAS in SJIA, which can theoretically be applied to AOSD. In our patient, features such as fever, hyperferritinemia, and thrombocytopenia with complex presentations upon admission further support the consideration of MAS as part of the diagnostic evaluation (the criteria are given in Table 3). Thus, AOSD is an inflammatory disease with multiorgan involvement, whereas MAS is a hyperinflammatory disease with multiorgan failure [15].

Treatment objectives in patients with AOSD include relieving the symptoms and signs, improving systematic inflammation, preventing end-organ damage, and reducing the long-term effects of treatment [16]. NSAIDs are the first-line treatment for those without systemic features, but they are not always effective. Corticosteroids are the mainstay of the treatments for those with systemic features. In those who are steroid refractory or with MAS features, targeted biological therapy with either IL-1 blockade (anakinra, canakinumab, or rilonacept) or IL-6 blockade (tocilizumab or sarilumab) can be used [17].

AOSD is self-limiting in most cases; however, in some patients, it can lead to severe and potentially fatal complications, including MAS, amyloidosis, disseminated intravascular coagulation (DIC), pulmonary arterial hypertension (PAH), thrombotic thrombocytopenic purpura (TTP), and diffuse alveolar hemorrhage. In this patient, recurrent hospital admissions revealed renal involvement and PAH, while other complications could not be conclusively excluded. These findings highlight the critical need for prompt detection and management of such complications to improve patient outcomes and reduce the risk of fatal consequences.

## Conclusions

AOSD poses a significant diagnostic challenge due to overlapping symptoms with other conditions and variable severity. This case demonstrates the complexity of AOSD diagnosis and management, with hallmark features including spiking fevers, a salmon-colored rash, and markedly elevated ferritin levels, reflecting systemic inflammation. These clinical indicators, alongside elevated inflammatory markers, should prompt suspicion of AOSD in cases of unexplained FUO. While AOSD is often diagnosed by exclusion, the presence of these typical signs can aid in early identification and prevent unnecessary investigations.
